# An agent-based model for household COVID-19 transmission in Gauteng, South Africa

**DOI:** 10.1371/journal.pone.0325619

**Published:** 2025-07-16

**Authors:** Folashade B. Agusto, Inger Fabris-Rotelli, Christina J. Edholm, Innocent Maposa, Faraimunashe Chirove, Chidozie W. Chukwu, David Goldsman, Suzanne Lenhart

**Affiliations:** 1 Department of Ecology and Evolutionary Biology, University of Kansas, Lawrence, Kansas, United States of America; 2 Department of Mathematics and Applied Mathematics, North-West University, Potchefstroom, South Africa; 3 Department of Statistics, University of Pretoria, Pretoria, South Africa; 4 Department of Mathematics, Scripps College, Claremont, California, United States of America; 5 Division of Epidemiology and Biostatistics, School of Public Health, Faculty of Health Sciences, University of the Witwatersrand, Johannesburg, South Africa; 6 Department of Mathematics and Applied Mathematics, University of Johannesburg, Johannesburg, South Africa; 7 Department of Mathematical Sciences, Georgia Southern University, Statesboro, Georgia, United States of America; 8 H. Milton Stewart School of Industrial and Systems Engineering, Georgia Institute of Technology, Atlanta, Georgia, United States of America; 9 Department of Mathematics, University of Tennessee, Knoxville, Tennessee, United States of America; Nanyang Technological University, SINGAPORE

## Abstract

Since the discovery of COVID-19 in Wuhan, China in 2019, close to seven million people have died from the infection. At the onset of the pandemic, many countries enacted stringent measures such as school and event closings in a bid to control and curtail the spread of the virus, leading to many within-household infections as people spent more time at home. This study develops an agent-based model (ABM) to gain insight into the impact of government COVID-19 mitigation guidelines and policy options on within-household and community COVID-19 infections in Gauteng, South Africa. Gauteng is the province in South Africa having the smallest land area, but it accounts for 25.8% of the country’s population. Agents are randomly assigned to cells on a 1000×1000 square grid varying according to Gauteng’s population density and household size distribution. We found that the percentage of within-household infections is higher in communities with smaller population densities, with the reverse being true for communities with larger population densities. Furthermore, as the agents’ movement activation rate increases, community-related infections increase, especially in communities with small population densities. Our study found an interesting phenomenon, observed for the first time: the existence of a movement activation threshold where the percentage and number of outside household infections overtake the percentage and number of within household infections when the activation rate increases. Lastly, our simulation results captured the two epidemic peaks experienced in Gauteng from March 30, 2020 to June 22, 2021 while varying quarantine violation and movement activation rates. Thus, the developed ABM can be used to exploit the implications of COVID-19 mitigation guidelines and policy options on household transmission to provide interesting insights.

## 1 Introduction

COVID-19, since its discovery in Wuhan, China in 2019 [22,38,45], has led to over five hundred million cases with over six million deaths [40]. At the start of the pandemic, many countries enacted numerous policies to try to control or contain the spread of the virus. Measures included school and event closings, as well as hard lockdowns on all social activities. The lockdown measures imposed by governments resulted in households as more favourable and important environments for transmission since activities outside the household were subject to enforced restrictions [4,27]. A prospective study in South Africa to evaluate SARS-CoV-2 burden and transmission showed that infected index cases transmitted the infection to about one in five susceptible household contacts [8]. In some parts of China and Europe, more than 78% of human-to-human transmission was found to occur, within families where mobility was reduced by at most 63% [25]; and a number of secondary cases came from confirmed patients within the households, as shown in [41]. The risk of transmission in households is affected by the household size since it is difficult to socially distance within households as contacts repeatedly interact during mealtimes and share facilities such as bedrooms, living rooms, and bathrooms [15,41]. In these instances, greater hours of exposure to an index patient, and greater closeness of exposure by physical contact or by sharing some common space are associated with increased COVID-19 risk [41]. Household transmissions are also affected by factors such as household income and the level of crowding in urban areas [15]. Moreover, there have been heightened increases in household transmissions with each successive SARS-CoV-2 variant [8], which are believed to contribute to successive waves. In South Africa, Cohen *et al*. [8] showed there was a high rate of SARS-Cov-2 infection in households with most infections being asymptomatic in individuals of all ages.

Households remain high-risk settings for transmission of COVID-19, and the understanding of the transmissibility of SARS-CoV-2 in such settings is important for informing infection prevention and control policies [4]. Previous models have explored the dynamics of household and community transmissions in relation to COVID-19 [13,14,16,23,42,43]. For instance, Gutiérrez-Jara *et al*. [14] considered a compartment model with movement between multiple locations in a community, focusing on home to ascertain behavioral impacts and policy changes during the pandemic. For households, Fyles *et al*. [13] proposed a branching process model with differing transmission rates within households, with an investigation into how to define a household and exploring the model with respect to contact tracing and epidemic extinction. Additionally, Hilton *et al*. [16] build upon the previous models adding in age structure to the household structure and formulating a model focusing on computational outcomes and management options.

In this study we focus on household and community COVID-19 outbreaks in Gauteng, a province of South Africa. South Africa has had the largest COVID-19 burden in Africa with over four million cases resulting in just over 100,000 deaths [35]. To curtail the outbreak, the South African government instituted five levels of control policies with Level 5 enacted from March 26, 2020. Level 5 was a hard lockdown, namely, only limited essential service individuals could leave their households. See Table 1 for summarized details of the policies and guidelines enacted during the time intervals $T_{1},\ldots ,T_{5}$. These time intervals were identified in Edholm *et al*. [10] as points of inflection which separate time periods where cumulative cases were increasing from the periods when the cumulative cases were decreasing; see Fig 1 in [10].

**Table 1:** Guidelines and policy changes for different alert levels in Gauteng. The time intervals $T_{1},\ldots ,T_{5}$ are separated by points of inflection identified in Edholm *et al*. [10]; these points separate time periods where the rate of cumulative cases was increasing from periods when the rate of cumulative cases was decreasing [10], Fig 1]. Note that the vaccinations of healthcare workers only started in February 2021 and the vaccinations only started in June 2021 for the elderly. Thus, the vaccinations roll-out did not have any impact on the model here.

	$T_{1}$	$T_{2}$	$T_{3}$	$T_{4}$	$T_{5}$
Duration	March 30, 2020 to July 12, 2020	July 13, 2020 to October 10, 2020	October 11, 2020 to December 27, 2020	December 28, 2020 to March 30, 2021	March 31, 2021 to June 6, 2021
Guidelines and policy changes	Hard lockdown, but gradually relaxed	Economic activities opened with increased mobility and crowding within and outside household	Restrictions on festive gathering	Vaccinations of health workers started	Vaccination of people aged 60 years or more and health workers continued

**Fig 1:**
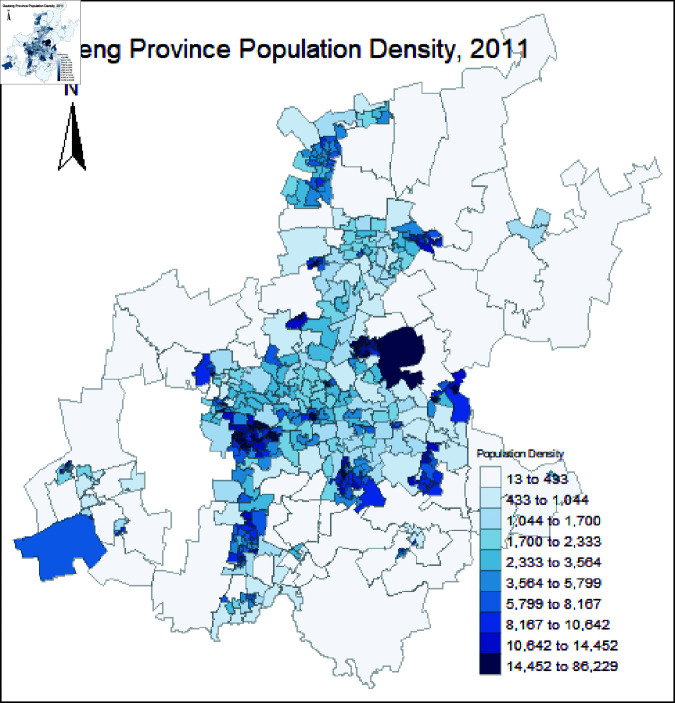
Map of Gauteng Population Density as of 2011 census data. The Gauteng basemap was obtained from DIV-GIS [9] and the population density map was generated using R version 4.4.1 [30].

The Gauteng province has the smallest land area of the provinces in South Africa, with $18178\;{\text{km}}^{2}$, covering less than 2% of the country. With a total population of 15,200,000, Gauteng, however, accounts for more than 25.8% of South Africa’s population [34], making it the country’s most-populous province. It contains several large population centers, including Johannesburg, Ekurhuleni Metropolitan, Soweto, and Tshwane Metropolitan. Fig 1 shows the distribution of Gauteng population density at the ward level as of 2011, using the latest census data available for South Africa.

This study uses an agent-based model (ABM) to represent the transmission of COVID-19 within households and the community. ABMs are mathematical models that can simulate and project epidemic trends, along with exploring the impact of different interventions [5,28]. They are also referred to as microsimulation models [29] or individual-based models [44], and can be regarded as a class of what are known as discrete-event simulations [21]. ABMs have been used to simulate the spread of SARS-CoV-2 in a variety of locations including the UK [12], Australia [32], and Singapore [20]. Some have also been developed to evaluate the impact of social distancing and contact tracing accounting for household and non-household contacts among other features [19,24,36]. Our newly developed model provides insight into how changes in guidelines and policies on closures and social distancing affect household and community transmission of COVID-19 in Gauteng province (with three mainly urban metropolitan districts and two mainly rural). The new model can also assist decisions of policy makers based on best available information while at the same time accounting for many uncertainties that accompany the epidemic in terms of transmission and infection dynamics. The rest of the paper proceeds as follows. Sect 2 gives an overview of the methods including transmission assumptions and implementation, Sect 3 describes the simulation, and Sects 4 and 5 present results and discussion, respectively.

## 2 Method

In this section, we describe the ABM’s structure by specifying: (i) the agents and their characteristics, (ii) agent interactions, (iii) disease transmission and status updates, and (iv) model implementation details. This study characterizes agents by their associated household (home) sizes, as well as disease status. The household sizes are structured according to the distribution of households in Gauteng as given by Statistics South Africa [37]. The distribution used is as follows: 25.5% of the population live in a one-person home, 41.1% live in 2–3 person households, 23.9% live in 4–5 person households, 9.4% live in 6-or-more person households, and 0.1% of the population are not classified; we assume these latter individuals are homeless. See Fig 3 for the household distribution.

An agent can be classified according to the following disease status: Susceptible to infection (*S*), exposed but not infectious (*E*), asymptomatic infectious (*A*), pre-symptomatic and symptomatic infectious (*I*), confirmed and quarantined (*Q*), quarantine violators (*Q*_1_), hospitalized (*H*), and recovered (*R*). These classes are the same classes in the unvaccinated model from [10] with the exception of the quarantined violators class (*Q*_1_), which represents individuals who break quarantine rules either due to mundane reasons like fatigue or to procure essential needs to maintain isolation [3].

The model captures the possible interactions of individuals within the household as well as the rest of the community. We assume agents interact with agents of the same household if they are at home or within the vicinity of their household (which we call the home radius *r*_*home*_). We also assume that when an agent leaves their home or their home radius, the agent is then part of the broader community.

**Fig 2:**
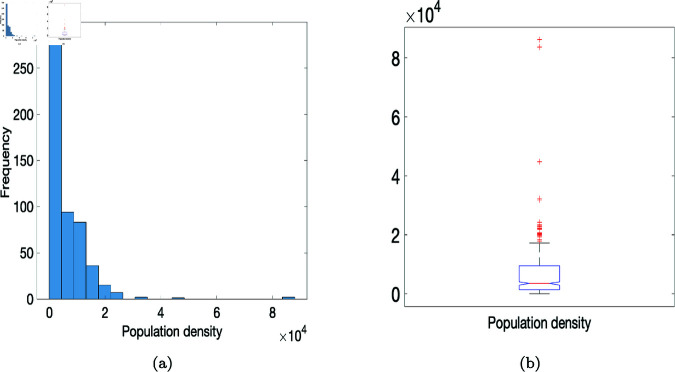
Distribution of Gauteng population density at the ward level in 2011. Data taken from [2]. (a) Gauteng population density; (b) Boxplot of Gauteng population density.

To initialize agent location we assign agents to households according to the population density of Gauteng (Fig 2). This province has 529 electoral areas called wards, with varying population densities, as shown in Fig 1. A standardized population density is used according to the size of each ward in ${\text{km}}^{2}$. We generate a sample from the population density distribution shown in Fig 2. Using the quartiles of the data we make use of four population density subsets (PD1, PD2, PD3, and PD4) from the minimum value to the largest outlier value, to investigate the effect of population density. We sample randomly from these four subsets (see Fig 1); and we draw from these population density subsets for the different scenarios in Sect 3.

**Fig 3:**
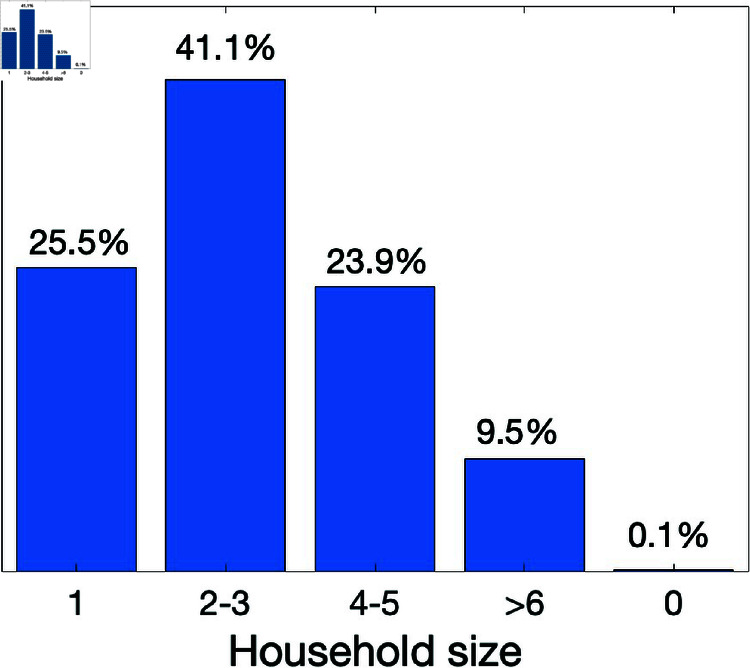
The Gauteng household size distribution [37] in which 25.5% of the population live in a one-person home, 41.1% live in 2–3 person households, 23.9% live in 4–5 person households, 9.4% live in more-than-6 person households, and 0.1% of the population are not classified.

In addition, agents are assigned to a household size matching the household-size distribution depicted in Fig 3. In this way, the modeled household structure is representative of the empirical pattern of household sizes and population density within the Gauteng province.

### Disease transmission and status updates

We assume agents can be symptomatic or asymptomatic, and they are randomly assigned to households according to the household distribution within Gauteng province. Over time as agents move and interact, we update their disease status; see Fig 5 for an overview of the ABM structure. In Fig 5, we layout the routine the ABM executes in the Python code for each agent, with the arrows mapping agent decisions to various action items based on yes/no responses (The code is available by contacting the corresponding author.).

**Fig 4:**
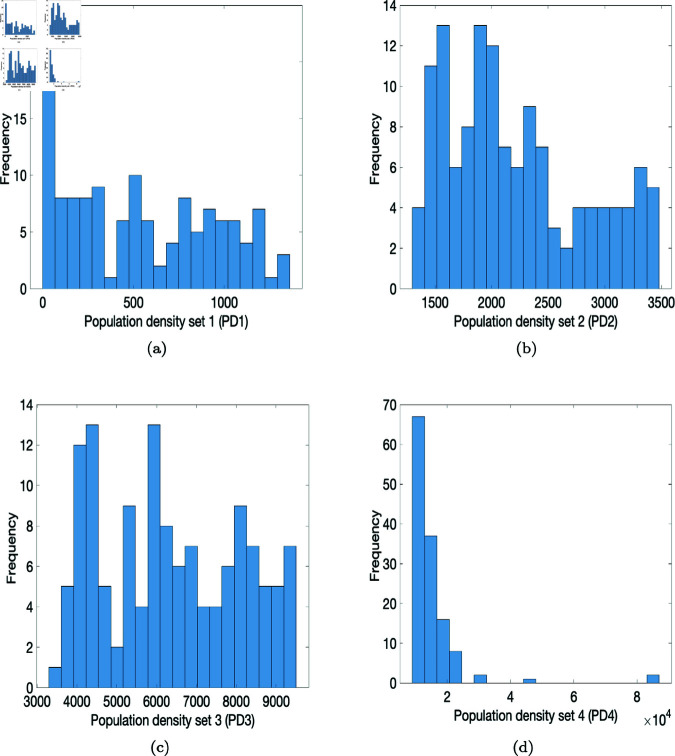
The distribution of Gauteng population density set intervals obtained from the four quartiles of the Gauteng population density boxplot. The population density distribution is shown in Fig 2. Population density set 1 (PD1) is obtained from the first quartile of the boxplot, and similarly population density sets 2, 3, and 4 (PD2, PD3, and PD4) are from the second, third, and fourth quartiles of the boxplot respectively.

**Fig 5:**
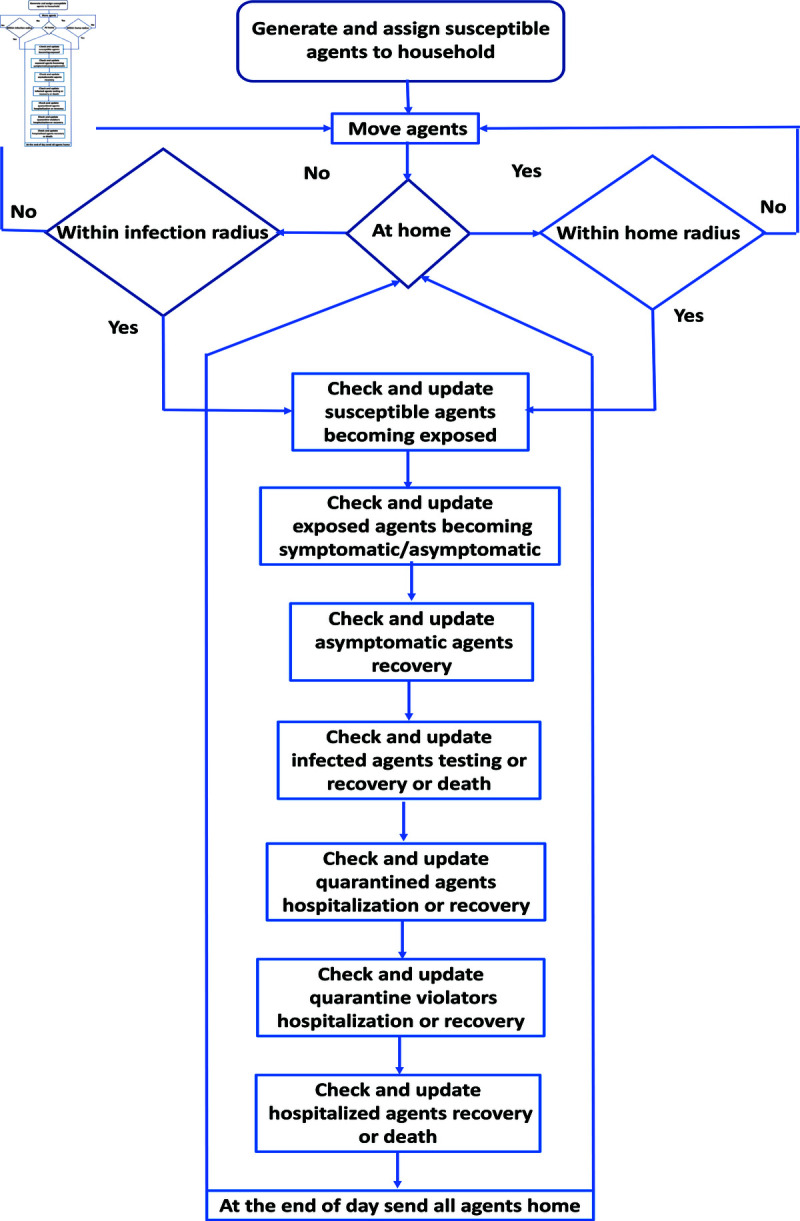
Flow diagram of the Python code process for agents characterization, and updating of their disease status. The arrows indicate directions following a decision shown in boxes and diamonds. In S1 Fig in S1 Appendix, we include a more granular diagram with specific probabilities for agent characterizations.

**Fig 6:**
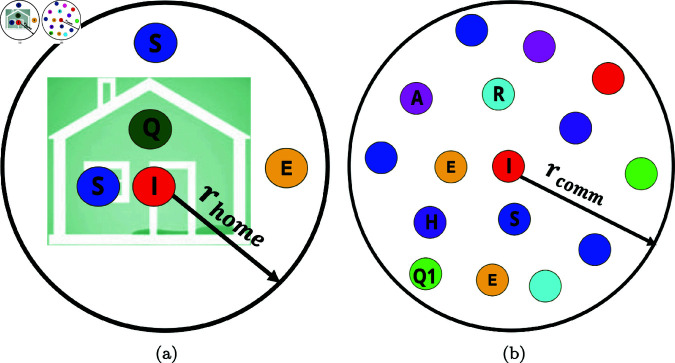
Infection radius is the distance from an infected agent in which it is possible for infection to occur. Blue represents susceptible agents, orange are exposed agents, magenta are asymptomatic agents, red are symptomatic agents, green are confirmed quarantined agents, neon green are quarantine violating agents, purple are hospitalized agents, and cyan are recovered agents. (a) Home radius of infectious agents; (b) Community radius of infectious agents.

When agents interact, the possibility of disease transmission is dependent on whether they are at home, within their home radius, or out in the community. It is possible for a susceptible agent at home to become infected if they are within *r*_*home*_ radius of an infectious member of their household (see Fig 6(a)), while a susceptible agent in the community can become infected if they are within an infectious radius *r*_*comm*_ of an infected agent; see Fig 6(b). For instance, in Fig 6(a), the two susceptible agents represented by blue can be infected by the infected agent represented by red since they are within *r*_*home*_ of the infected agent. Similarly, in Fig 6(b), the susceptible agents within *r*_*comm*_ of the infected agent can become infected; however, the other susceptible agents outside this radius cannot be infected. These infection radii also apply to susceptible agents within the home or community radii of asymptomatic infectious agents.

Following the approach of Keeling and Rohani [26], we convert the rates in Edholm *et al*. [10] to probabilities as follows. The probability of a susceptible agent getting infected at home is dependent on the risk of infection at home and is given by


$$1-\exp(-\frac{\beta \times \lambda_{home}\times \Delta t}{\text{household size}}),$$

where the quantity $\lambda_{home}$ is the total number of infected (symptomatic or asymptomatic) agents within the home radius (*r*_*home*_) of a susceptible agent who are members of the agent’s household (see [11,39] for similarly defined force of infection). The parameter β is infection rate of COVID-19 to a susceptible agent and Δt is the increment of time, using a day here.

The probability of getting infected within the community is given by


$$1-\exp(-\frac{\beta \times \lambda_{comm}\times \Delta t}{\#\text{ of agents in the community}}),$$

where the quantity $\lambda_{comm}$ is the total number of infected (asymptomatic or symptomatic) agents within the infection radius *r*_*comm*_ of a susceptible agent in the community. Once susceptible agents are infected they become exposed, and their status is then updated from *S* to *E*.

It is important to note that if the radius is too small, infections will happen more quickly and if the radius is too large infections will happen more slowly. We tried different values of both home radius (*r*_*home*_) and infection radius (*r*_*comm*_) before fixing the home and infection radii to values of 20 and 15 grid cells for the different simulation runs. Considering the simulation was conducted on a 1000×1000 grid, larger and smaller values for these radii resulted in unrealistic simulations in terms of spread and time to spread. For applications of this ABM, the radii should be chosen in a data-driven manner.

An exposed agent with disease status *E* transitions to asymptomatic infectious class *A* with probability $1\;-\;e^{-\alpha \varepsilon \Delta t}$, or to the symptomatic class *I* with probability $1\;-\;e^{-\alpha (1-\varepsilon )\Delta t}$. Once in either the asymptomatic or symptomatic class, the disease status gets updated to *A* or *I* respectively. The parameters α and ε are constants defined in Table 2.

**Table 2:** Parameters and descriptions of the ABM model.

Parameter	Biological Meaning	
β	transmission rate	per day
1/α	length of exposure period	days
ε	proportion of asymptomatic out of infectious individuals	unitless
μ	death rate for symptomatic, quarantined, and	per day
	quarantine violating agents	
$\mu_{H}$	death rate for hospitalized agents	per day
σ	quarantine violation rate	per day
κ	COVID-19 testing/confirmed rate	per day
ρ	hospitalization rate for quarantined agents	per day
$\gamma_{1}$	recovery rate for asymptomatic agents	per day
$\gamma_{2}$	recovery rate for symptomatic agents	per day
$\gamma_{3}$	recovery rate for quarantined agents	per day
$\gamma_{4}$	recovery rate for quarantined violating agents	per day

An asymptomatic agent, *A*, recovers with probability $1\;-\;e^{-\gamma_{1}\Delta t}$, and their status is updated to recovered status *R*. A symptomatic agent, *I*, recovers with probability $1\;-\;e^{-\gamma_{2}\Delta t}$ or they get tested and confirmed to have COVID-19 with probability $1\;-\;e^{-\kappa \Delta t}$. If agents test positive, they transition into the quarantine class and their status is updated to *Q*. Quarantined agents recover with probability $1\;-\;e^{-\gamma_{3}\Delta t}$ or they violate the quarantine rules with probability $1\;-\;e^{-\sigma \Delta t}$. Quarantined violating agents recover with probability $1\;-\;e^{-\gamma_{4}\Delta t}$. Agents that are quarantined or violating quarantine rules are hospitalized with probability $1\;-\;e^{-\rho \Delta t}.$

Lastly, symptomatic, quarantined, and quarantine-violating agents die with the probability $1\;-\;e^{-\mu \Delta t}$, while the hospitalized agents die with the probability $1\;-\;e^{-\mu_{H}\Delta t}.$ These parameters are summarized in Table 2 and a transition diagram showing the interact of the agents in the population is given in S1 Fig in S1 Appendix. This figure show how a susceptible individual becomes infected following their assignment to a household.

## 3 Model implementation

The model is implemented in Python 3.8 on a 1000×1000 square grid amounting to 1,000,000 cells. Agents are randomly assigned to these cells according to the Gauteng population densities (PD1, PD2, PD3, and PD4) and household size distribution shown in Fig 3. We make use of the parameter values as obtained in Edholm *et al*. [10] and repeat them here in Table 3.

**Table 3:** Parameter values (see Edholm *et al*. [10] for details of the estimation).

Parameters from Literature					
Symbol	Value	Symbol	Value	Symbol	Value
α	0.25 [17,33]	ε	0.3 [6]		
$\gamma_{1}$	0.14 [7,17]	$\gamma_{2}$	0.14 [7,17]	$\gamma_{3}$	0.1 [17,31]
Constant Estimated Parameters					
ρ	0.14153	μ	0.01414	$\mu_{H}$	0.09376
		$\gamma_{4}$	0.06669		
Estimated Parameters that Change over Time					
Symbol	*T*_1_	*T*_2_	*T*_3_	*T*_4_	*T*_5_
β	0.53855	0.16862	0.27703	0.19947	0.41912
κ	0.73861	0.33931	0.09782	0.50617	0.49855

These parameters were obtained for the five time periods $T_{i},\;i=1,\ldots ,5$ (separated by inflection points of cumulative cases of COVID-19 infections), depicted in Table 1 regarding the government guidelines and policy changes for different national alert levels. These inflection points were estimated by combining the weekly cumulative confirmed cases for Gauteng and the interpretation of the government policy changes and individual behaviors (see Fig 1 in Edholm *et al*. [10]). These time points separate portions of the cumulative cases data, where the number of cases are rising (*T*_2_ and *T*_4_) from the portions of the data where the cumulative cases are decreasing ($T_{1},\;T_{3},$ and *T*_5_). The data used in Edholm *et al*. [10] was obtained from the Data Science for Social Impact research group at the University of Pretoria [1,18].

### Simulation

In this study, we keep track of the number of symptomatic and asymptomatic agents, as well as where the infections occur, either at home or within the community. The model dynamics are explored through Monte Carlo simulations, where each scenario of interest is simulated 100 times. We initialize and simulate the model for the different time periods $T_{i},\;i=1,\ldots ,5$, using as the initial numbers of symptomatic and asymptomatic agents the corresponding numbers of infected individuals obtained from Edholm *et al*. [10]; see Table 4. These numbers were first converted to their population density equivalent standardized by dividing the population size by the land area.

**Table 4:** Initial asymptomatic ($A_{0}$) and symptomatic ($I_{0}$) agents obtained from Edholm *et al*. [10] during time periods $T_{1},\ldots ,T_{5}$.

	*A*_0_	*I*_0_
*T*_1_	2331	1174
*T*_2_	19798	10843
*T*_3_	576	308
*T*_4_	17739	24190
*T*_5_	661	258

The simulation starts with the agents moving out of their assigned homes according to a given movement activation rate. This rate is the speed with which the agent move around; normally, speed is calculated as distance traveled/time. For simplification we fixed this speed for all the agents. The agents then interact over the course of a day either with agents in their households or other agents in the community. At the end of the day, the agents return home and the simulation repeats for each time frame $T_{1},\ldots ,T_{5}$. See the model flow diagram in Fig 5.

## 4 Results

**Fig 7:**
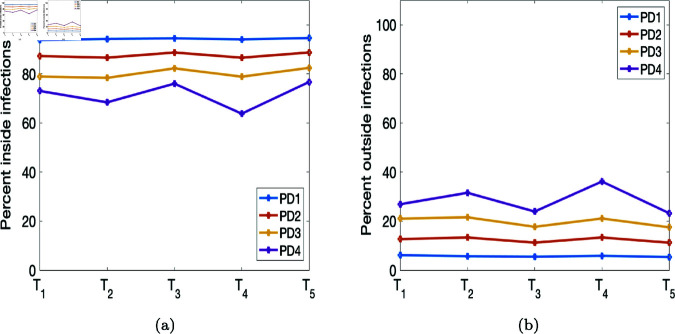
Percentages of infections inside and outside of households drawn from the four population density sets as depicted in Fig 4.

**Fig 8:**
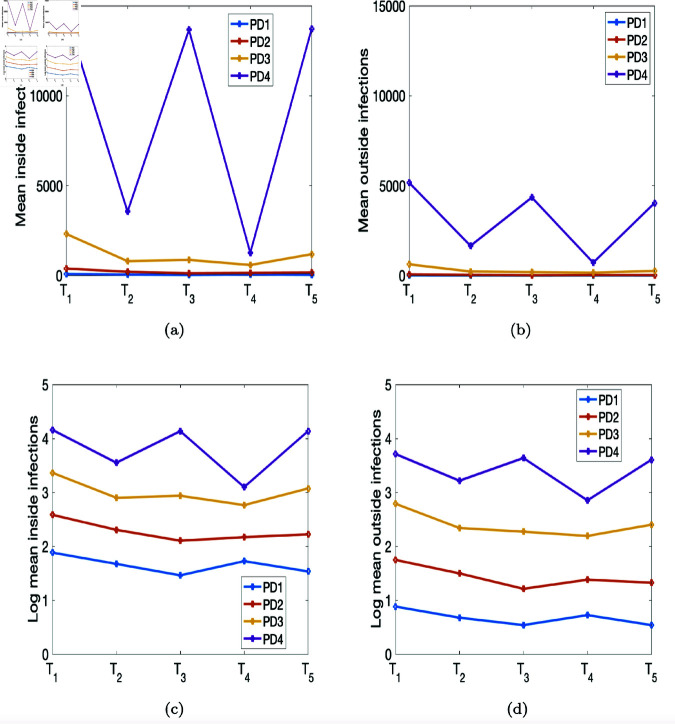
Mean numbers of infections inside and outside of households drawn from the four population densities PD1–PD4 depicted in Fig 4. (a) and (b) Mean numbers of infections within and outside the household. (c) and (d) Logs of mean numbers of infections within and outside the household.

**Fig 9:**
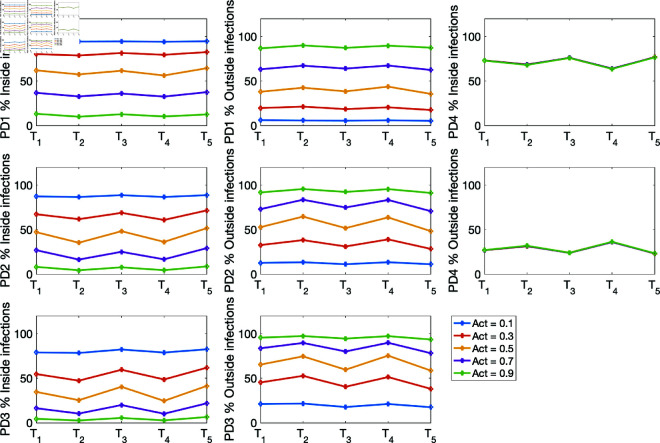
PD1–PD4 percentages of inside and outside infections with different movement activation values 0.1, 0.3, 0.5, 0.7, and 0.9 with σ=0.6. The means are drawn from each of the four population densities set PD1–PD4 depicted in Fig 4.

**Fig 10:**
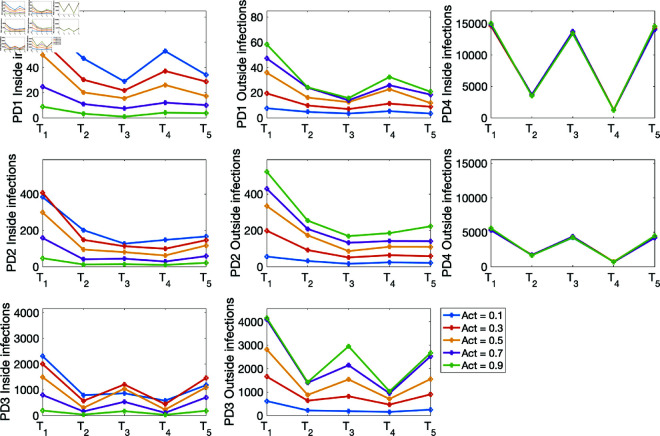
PD1–PD4 mean numbers of inside and outside infections with σ=0.03 for movement activation rates of 0.1, 0.3, 0.5, 0.7 and 0.9.

**Fig 11:**
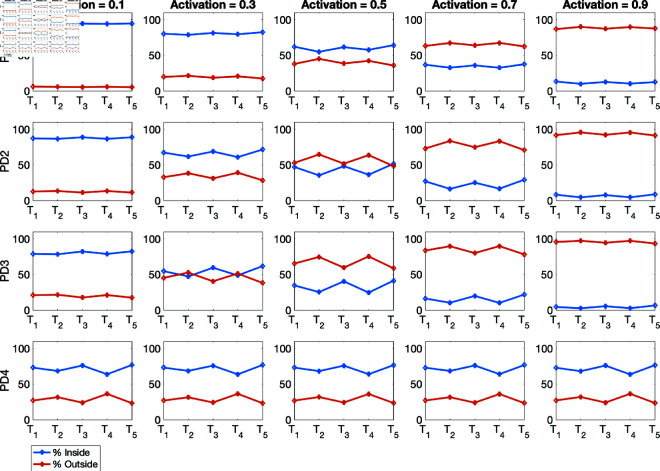
PD1–PD4 percentages of inside and outside infections with σ=0.03 for movement activation rates of 0.1, 0.3, 0.5, 0.7, and 0.9. Observe the presence of a movement activation threshold for PD1, PD2, and PD3 where the percentage of outside household infections overtakes the percentage of within household infections when the activation rate increases.

**Fig 12:**
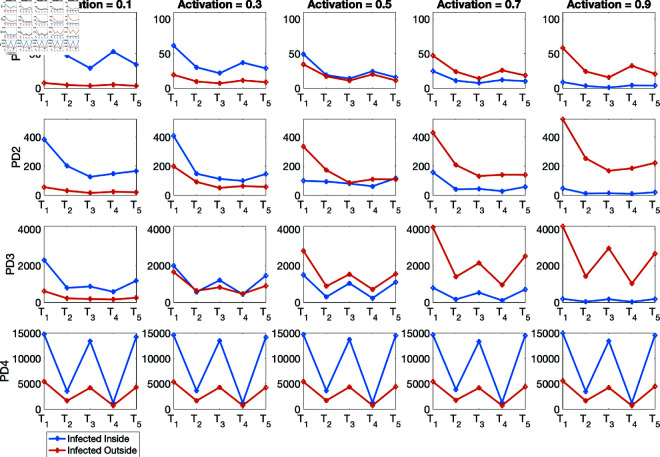
PD1–PD4 mean numbers of inside and outside infections with σ=0.03 for movement activation rates of 0.1, 0.3, 0.5, 0.7, and 0.9. Observe the presence of thresholds for PD1, PD2, and PD3 where within household switches from increasing to decreasing as the movement activation rates increase.

**Fig 13:**
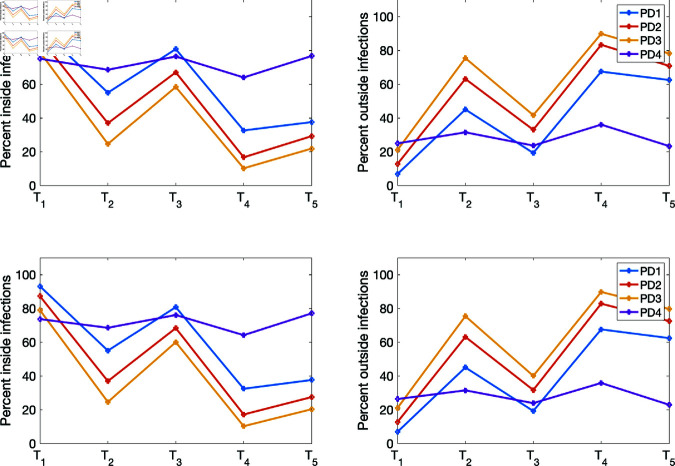
Percentages of infections within and outside the household for two scenarios where movement and quarantine violation rates are varied to mimic changes in policies during $T_{1}$–$T_{5}$ using values in Table 6. Top row: Scenario 1 - Percentages of within and outside infections while varying σ and the movement activation rates. Bottom row: Scenario 2 - Percentages of within and outside infections with constant σ while varying the movement activation rates.

**Fig 14:**
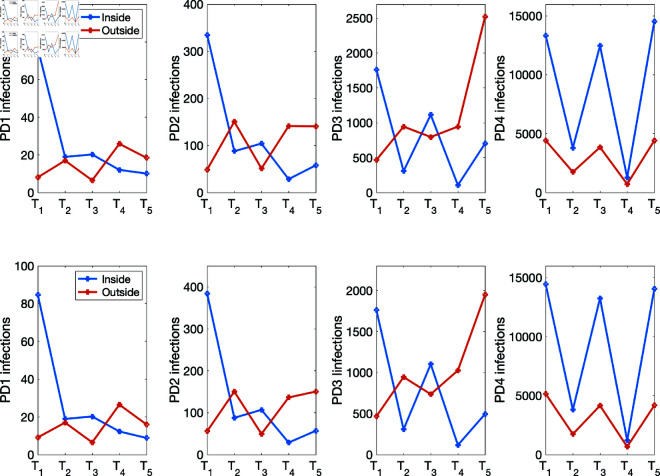
Number of infections within and outside the household for two scenarios where movement and quarantine violation rates are varied to mimic changes in policies during $T_{1}$–$T_{5}$ using values in Table 6. Top row: Scenario 1 - Percentages of within and outside infections while varying σ and the movement activation rates. Bottom row: Scenario 2 - Percentages of within and outside infections with constant σ while varying the movement activation rates.

For each of the various scenarios and for each of the different simulation periods ($T_{i},\;i=1,\ldots ,5$), we record the percentage of within-household infections and infections that occur outside the household. We also record the total number of infections; for each scenario and time frame, this is the sum of within- and outside-household infections during that time frame. We also record the sample mean and variance over the 100 independent replications for the different time periods. We simulated the model under the following scenarios:

(i) Using the baseline parameter values in Table 3. The results are given in Fig 7 for the percentage of infections inside and outside of households, and Fig 8 for the mean numbers of infections inside and outside of households for the four population density cases PD1–PD4.
(ii) Varying the movement activation rate with values set as 0.1, 0.3, 0.5, 0.7, and 0.9, with σ=0.6 to mimic changes in policies during $T_{1},\ldots ,T_{5}$ time periods. The results are given in Fig 9 for the four population-density cases PD1–PD4, each of which depicts the percentages of inside and outside infections for the different movement activation values. Fig 10 shows the corresponding sample mean plots of the numbers of inside and outside infections for these movement activation rates.
(iii) Varying quarantine violation and movement activation rates. The plots of PD1–PD4 percentages of inside and outside infections with σ=0.03 for movement activation rates of 0.1, 0.3, 0.5, 0.7 and 0.9 are shown in Fig 11. Fig 12 illustrates the corresponding sample mean plots of the numbers of inside and outside infections for PD1–PD4.
(iv) Lastly, we simulate two scenarios where movement and quarantine violation rates are varied to mimic changes in policies during time frames T1–T5. Fig 13 shows the percentages of infections inside and outside the household, while the numbers of infections are given in Fig 14.

Next, we describe in detail the results of these scenarios in Figs 7–14 below.

Fig 7 depicts the percentages of infections within and outside the households. We see from Fig 7(a) that the percentages of infections from within households in the less-dense areas are high compared to the highly dense region. Conversely, we see from Fig 7(b) that outside household infections are higher in the more-densely populated regions compared to the less-dense areas. In the simulation underlying Fig 7, we set the movement activation rate to 0.1 and the quarantine violation rate σ=0.03; in other scenarios below, we vary the movement activation rate. However, when the movement activation rate is higher, we see fewer infections within the household (see Fig 9). Furthermore, the trajectories of both the percentages and numbers of infections within and outside the household mirror each other.

Fig 8(a) and 8(b) depict the mean numbers of infections within and outside the household. The mean numbers of infections from PD4 are larger than those of the other population densities, and so we log-transform those mean numbers of infections; see Fig 8(c) and 8(d) for the log-transformed figures. In Fig 8(c) and 8(d), we observe that the logs of the mean numbers of infections for both within and outside the households are smallest for the less-populated regions (PD1 and PD2), unlike what we see for the highly dense (PD3 and PD4) areas of the province. In addition, the mean numbers of agents both within and outside the households are high for the time periods *T*_1_, *T*_3_, and *T*_5_ and low for the time periods *T*_2_ and *T*_4_, as we observed from the cumulative cases data depicted in Fig 4 obtained from Edholm *et al*. [10].

Moreover, in Table 5 we provide the standard deviation (SD) values obtained for 100 simulations runs carried out for PD1–PD4 inside and outside household infections. We observe in the table lower variation in the number of infections in the less-dense areas (PD1 and PD2). On the other hand, the dense areas (PD3 and PD4) have relatively larger variation in the numbers of infections for both within and outside the households.

**Table 5:** Standard deviations of the number of inside and outside household infections based on 100 replications of PD1–PD4 simulations.

	*T*_1_	*T*_2_	*T*_3_	*T*_4_	*T*_5_
PD1 SD Inside	67.59	41.80	29.86	38.78	35.55
PD2 SD Inside	153.58	137.60	78.12	119.49	111.21
PD3 SD Inside	954.88	515.16	604.64	375.97	790.04
PD4 SD Inside	2757.57	1350.36	3240.41	415.44	2542.51
PD1 SD Outside	7.16	4.30	3.01	3.41	2.88
PD2 SD Outside	32.92	23.52	12.73	18.47	16.26
PD3 SD Outside	329.20	151.12	150.07	112.10	199.93
PD4 SD Outside	1240.28	534.10	1425.20	235.23	1082.08

### Varying the movement activation rate

Next, we explore the effects of varying the movement activation rates from 0.1 to 0.9 with a step-size of 0.2. Fig 9 reveals a decrease in percent infections within households and an increase in percent infections outside as the movement activation rate increases in the PD1–PD3 population scenarios. The percent infections within and outside households for the population density PD4 stayed the same, but the infections within the household are about 70% for all the $T_{1},\ldots ,T_{5}$ time frames, and infections from outside are about 30%. This agrees with estimates from the literature on household COVID-19 infections [15,25,41]. Fig 10 shows the infection burden for each of the population densities as the movement activation rate increases from 0.1 to 0.9 by 0.2.

Fig 11 shows the percentages of infections inside and outside for population densities PD1–PD4 for movement activation rates 0.1, 0.3, 0.5, 0.7, and 0.9, when the quarantine violation rate is set at σ=0.03. Fig 11 clearly illustrates the surprising presence of a movement activation threshold value for population densities PD1–PD3 when the percentages of within and outside infections are the same. Away from this movement activation threshold value we see a switch from increase to decrease in the percentage of infections within household and a switch from decrease to increase in the percentage of infections outside as the movement activation rate increases from 0.1 to 0.9 by a step-size of 0.2. For PD1, this switch is observed when the movement activation rate changes from 0.5 to 0.7. For PD2, the switch occurs at movement activation rate 0.3 to 0.5; and for population density PD3 the threshold switch is at movement activation rate 0.3. Interestingly, this threshold switch was not observed for PD4; but going by the switch patterns for the other population densities that occur at comparatively low movement activation rates with an increase in population density, it is likely that the switch would have occurred at a very low activation rate for PD4.

Fig 12 plots the numbers of infections within and outside the household. This figure also shows the presence of a threshold when the numbers of inside and outside infections are the same. After the threshold the numbers of infected individuals switch from either increasing to decreasing (for within household infections) or decreasing to increasing (for outside household infections).

### Varying quarantine violation and movement activation rates

In these next scenarios we focus on and evaluate different what-if scenarios. First, we exploit changes in quarantine violation and movement activation rates to mimic changes in policies during time frames $T_{1},\ldots ,T_{5}$. For instance, during *T*_1_, there was a hard lock down; in this case, we assume that movement in the community and the quarantine violation rate are very low, so we set σ=0.03 and the activation rate to 0.1. However, during *T*_4_ and *T*_5_, health workers and people aged 60 years and above were vaccinated; and since there was no restriction on movement during these times, we set σ=0.6 and activation rate = 0.7. In another scenario we keep the quarantine violation rate constant (σ=0.3) and vary the movement activation rate. See Table 6 for the values used for the different scenarios designated as 1–4. The quarantine violation rate is the transition rate from the *Q* class to the *Q*_1_ class due to vital or non-vital reasons [3].

**Table 6:** Different scenarios where movement and quarantine violation rates are varied to mimic changes in policies during time periods $T_{1},\ldots ,T_{5}$. In the first scenario, movement and quarantined are varied, setting the movement activation rate as 0.1, 0.3, 0.5, 0.7, 0.7, and quarantine violation rate (σ) as 0.03, 0.3, 0.03, 0.6, 0.6. In the other scenarios (2–4), quarantine violation rates (σ) are held constant at 0.03, 0.3, and 0.6 while varying the movement activation rate.

Scenarios	Quarantine Violation Rate (σ)	$T_{1}$	$T_{2}$	$T_{3}$	$T_{4}$	$T_{5}$
1	Variable	0.03	0.3	0.03	0.6	0.6
2	Constant	0.03	0.03	0.03	0.03	0.03
3	Constant	0.3	0.3	0.3	0.3	0.3
4	Constant	0.6	0.6	0.6	0.6	0.6

Fig 13 reports results corresponding to scenarios 1–4 given in Table 6. The PD1–PD3 results from all of scenarios 1–4 reveal a high percentage of infections within the household during *T*_1_ and a low percentage of infections outside the household when the movement activation and quarantine violation rates are set to 0.1 and 0.03, respectively. During *T*_2_ we increased the movement activation rate from 0.1 to 0.5, and the quarantine violation was increased from 0.03 to 0.3; for this scenario, we found that the percentage of infections outside the household increased and the percentage of within household infections decreased. Similarly, during *T*_3_, the percentage of within household infections increased while outside infections decreased when the movement activation rate decreased to 0.3 from 0.5 and the quarantine violation had also decreased from 0.3 to 0.03 during *T*_3_. During period *T*_4_, the percentage of infections outside the household increased as the movement activation rate increased from 0.3 to 0.7, while the percentage of within household infections decreased. We observe an increase in the percentage of within household infections during period *T*_5_ and a reduction in outside infections while the movement activation and quarantine violation rates remained unchanged from period *T*_4_; these results are due to the changes in the transmission and quarantine rates between periods *T*_4_ and *T*_5_ (see Table 3). The solution profile of the trajectory for PD4 remained relatively the same as those in Fig 9. The solution profiles for all the population densities were similar for constant quarantine violation rate σ with variable movement activation rate, i.e., scenarios 2–4 in Table 6.

Fig 14 shows the number of infections within and outside the home as we vary the movement activation and quarantine violation rates to match the policies implemented during times *T*_1_–*T*_5_. In Table 7, we compare the results of the four scenarios using various fixed and variable quarantine violation rates. The solution profiles in Fig 14 look relatively similar. This is because in these simulation runs the effect of the different quarantine violation rates (σ=0.03, 0.3, and 0.6) on the number of infections across the different population densities is relatively small. Summing the numbers of within and outside household infections across PD1–PD4 and comparing the outcomes in Table 7 we observed that for constant quarantine violation rate with variable movement activation, infections within and outside the household increase as more agents violate the quarantine rules. With variable quarantine violation rates the total number of infections is higher than total infections from the lowest quarantine violation rate (σ=0.03) and lower than that from the highest quarantine violation rate (σ=0.6). We would expect infections to increase as we increase the quarantine violation rate while also varying the movement activation rates.

**Table 7:** Outcomes of different scenarios when movement and quarantine violation (σ) rates are varied to mimic changes in policies during time periods $T_{1}$–$T_{5}$. In the first scenario movement activation and quarantine violation rates are varied setting the movement activation rate as 0.10, 0.3, 0.5, 0.7, 0.7, and quarantine violation rate as 0.03, 0.3, 0.03. 0.6, 0.6. In other scenarios quarantine violation rates are held constant at 0.03, 0.3, and 0.6, respectively, while varying the movement activation rates.

Scenarios	Quarantine Violation Rate (σ)	Inside	Outside
1	Variable	50183	21464
2	Constant (σ=0.03)	47152	19718
3	Constant (σ=0.3)	51376	21678
4	Constant (σ=0.06)	53283	23045

## 5 Discussion and conclusions

### Discussion

In this work, we developed an agent-based model for COVID-19 using parameter values obtained from Edholm *et al*. [10] for COVID-19 case data from Gauteng, a province of South Africa. Gauteng is the most-populous region of South Africa with 529 electoral wards, including the cities of Johannesburg, Ekurhuleni, and Tshwane. It comprises has a total of $18178\;{\text{km}}^{2}$, the smallest land area of the provinces in South Africa.

In this study, we characterize agents by household sizes and disease status. We structured agents’ household types according to the distribution of households in Gauteng [37]. Gauteng’s household size distributions are as follows: 25.5% of the population live in a one-person home, 41.1% live in 2–3 person households, 23.9% live in 4–5 person households, 9.4% live in 6-or-more person households, and 0.1% of the population are not classified. Recall that the household size distribution is given in Fig 3.

First, our study shows that both the percentages and average numbers of infections within and outside the household mirror each other; meaning if infection is, say, high for within household, it will be low for outside infection and so forth. Furthermore, our study shows that the percentages of within household COVID-19 infections in lower density communities (such as PD1 and PD2) are high while infections from outside the households are low, unlike the case for highly dense communities (such as PD3 and PD4); in those latter cases, within household infections are low while outside households infections are high when agent movement activation is low—although the overall numbers of infections in highly dense communities are much higher (see Figs 7 and 9). Corresponding to the differences that we see between PD1 and PD3, Liu *et al*. [23] suggests that dissimilarity in household size distribution can lead to significant differences in COVID-19 incidence between two regions. Furthermore, we observed that as more agents move around, community related infections increase in communities with small population densities. These results align with results observed in some parts of China and Europe by Madewel in [25] where within family transmissions can be more than 78% when mobility was reduced by at most 63%. We also observed in Figs 7 and 9 that within household infections were between 50% to 80% among all the population densities. However, within household infections were about 70% in PD4, the highly dense population. Note that implementation of a stay-at-home policy and restriction of movement could reduce infection. Our model inherently includes a stay-at-home policy and restriction of movement in the different time periods, reflecting changes in within and outside infections. The model of Yuan *et al*. [43] showed that when a stay-at-home policy was implementation in Canada, the contact rate outside the household fell by 39% leading to a decrease in the effective reproductive number from 3.56 to 0.84.

From March 30, 2020 to June 22, 2021, Gauteng experienced two waves of COVID-19 with peaks during the *T*_2_ and *T*_4_ time periods (see Fig 4 in Edholm *et al*. [10]). Our simulation results for the percentages of infections shown in Figs 7, 9, and 11 were able to capture these peaks for outside infections as the percentages of infections were higher during these time periods. But the mean numbers of infections in Figs 8, 10, and 12 for both within and outside household infections did not reflect these peaks. This is due to the fact that we kept the quarantine violation and movement rates the same for the simulation runs. However, when we varied the quarantine violation and movement activation rates to mimic changes in government policies and people’s behavior, we were able to capture these two peaks for the percentage of outside infections (see Fig 13). And the percentage of infections was higher during *T*_4_ when the second peak occurred compared to *T*_2_ when the first peak occurred. Indeed, as observed in Edholm *et al*. [10], the number of infections at the second peak was higher than the first peak.

**Fig 15:**
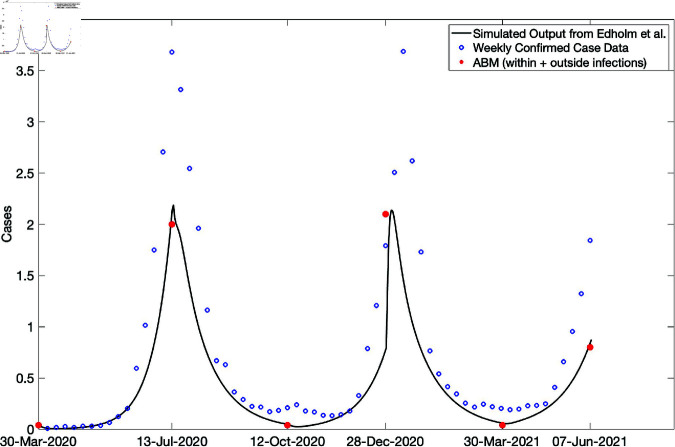
Numbers of infections from weekly confirmed cases, with simulated output from Edholm *et al*. and the sum of within and outside the household infections from PD1–PD4 during $T_{1}$–$T_{5}$. The behavior of the red dots matches the trajectory of the data and the simulation from Edholm *et al*. The value of the red dot at $T_{4}$ is higher than the value at $T_{2}$ where the solid black curve represents the simulated results and the blue dots represent reported data.

Furthermore, our simulation results depicted in Fig 14 for the mean numbers of infections showed the presence of these two peaks, especially for the PD1, PD2, and PD3 population densities. The mean number of outside infections in PD1 was clearly higher during *T*_4_ than *T*_2_. This was not the case for PD4 as the mean number of infections was a bit lower during *T*_4_ and *T*_2_. A number of factors might be responsible for this, for instance, the home and community radii used might not be suitable for the large population density and might require adjusting to reflect the dynamics for such large populations. Note that when we sum the mean numbers of within and outside household infections across the four population densities, the behavior of the simulation results matches the trajectory of the raw data and that of the Edholm *et al*.; see Fig 15. This figure captures the overall behavior of the pandemic for the given time and location. At the peaks uncertainty on true case numbers is higher than in the rest of the time periods due to significant hospital and testing capacity issues in South Africa. Also we observed in Figs 11 and 12 the absence of a switching threshold for the population density which might be attributed to the size chosen for these two radii. More work is required to explore the effect of these radii on the population densities.

In Figs 11 and 12, our simulation results captured an interesting phenomenon where the percentages (and numbers) of infections within and outside the household are the same. This indicates the presence of a threshold where the infections switch from either increasing to decreasing or from decreasing to increasing with reducing movement activation rate as the population density increases. To the best of our knowledge, this is the first time such an observation has been made and more needs to be done to explore its implications for disease transmission and control.

Our simulation results illustrate the impact of quarantine violation on both within and outside household infections. As expected, we saw in Table 7 that as more people violate the quarantine rules, infection increases and there are more infections within and outside the household. We would also expect infection to increase as we increase the quarantine violation rate with variable mobility rate. This agrees with results in Agusto *et al*. [3] that showed an increase in the COVID-19 epidemic curve as the quarantine violation rate was doubled, and the epidemic peaked earlier. Like the results in Agusto *et al*. [3] which showed the possibility of multiple waves of infections due to non-altruistic behavior of the infectious isolated individuals, our developed agent-based model using Gauteng COVID-19 related parameters was able to capture the two waves of infections experienced in the province during that time period.

### Conclusions

In this study we have developed an agent-based model for COVID-19 using parameter values estimated in Edholm *et al*. [10] for COVID-19 case data from Gauteng province of South Africa. We randomly assign agents to cells on a 1000×1000 square grid according to Gauteng’s population densities and household size distribution [37]; see Fig 2 for Gauteng’s population density at the ward level, and Fig 3 for the household size distribution. Our goal was to provide insight into how changes in government guidelines and policies on closures and social distancing affect household and community transmission of COVID-19 in Gauteng using an agent-based model. Our results aligns results observed in some parts of the world like Canada, China, and parts of Europe. We summarize below results from this study as follows:

1. The percentage of inside house infections is higher in communities with smaller population densities leading to lower outside infections. The reverse is true for communities with larger population densities;
2. As more agents move out and about due to an increase in the movement activation rate, community related infections increase, especially in communities with small population densities;
3. There exists a movement activation threshold where the percentage and number of outside household infections overtake the percentage and number of within household infections when the activation rate increases;
4. As quarantine violation and movement activation rates increase, infections within and outside the household increase;
5. The simulation results for the percentage of infections outside the household captured the two epidemic peaks experienced in Gauteng from March 30, 2020 to June 22, 2021 and mirrored the trajectories for within household infections.

Thus, our study shows that an agent-based model can be used to exploit the implications of government COVID-19 mitigation guidelines and policy options with interesting and surprising results. For instance, we observed the presence of a movement activation threshold value where the percentages of within and outside infections are the same, but away from this movement threshold value the percentage of infections within and outside household switches direction from increasing to decreasing (or from decreasing to increasing) as the movement activation rate increases. In the future, we will quantify the functional relationship between movement activation threshold, percent inside and percent outside, and time. We believe this quantification will make our result more robust.

Our developed model has a couple of limitations, which we hope to address in subsequent work. For instance, in accounting for the heterogeneity in the distribution of Gauteng population density, we grouped the province according to the size of the wards without any interaction between the groups. However, the distribution of Gauteng population density is not clustered by size but is rather heterogeneous in nature (see Fig 1), and there is movement and interaction of individuals across each ward of the province. While agents are assigned to households with different sizes, we did not track the number of infections from specific sizes of these households. Although this is not a limitation per se, such insight will reinforce previous knowledge about large household sizes and infections. The mechanism for the incorporation of population density is one of sampling from the distribution of densities across the Gauteng province. An interesting adaptation could be to incorporate the spatial information shown in Fig 1 into this strategy to account for the heterogeneous nature. Another limitation that could be of importance is the use of the 2011 South African Census data. There is unfortunately no updated Census since 2011. However, it is not expected that the distribution of the densities would differ significantly since 2011. It would be easy to replicate the study with updated data.

Another aspect of our future work will be to extend the mechanism developed in this study to study the different COVID-19 variants and other diseases that fits this disease profile. Although, this will be with great care since infectious disease models follow the natural history of infection.

## Supporting information

S1 Appendix Model flow diagram. (TEX)

S1 Fig From Fig 5, a flow diagram describing the interactions between the compartments/agents. With this diagram we display the specific probabilities used by the Python code for the ABM.
(TIFF)
