# Supplementary figures and images for: An agent-based model for household COVID-19 transmission in Gauteng, South Africa
Source: PLoS One. 2025 Jul 16;20(7):e0325619. doi: 10.1371/journal.pone.0325619 (PMC12266425; doi:10.1371/journal.pone.0325619)

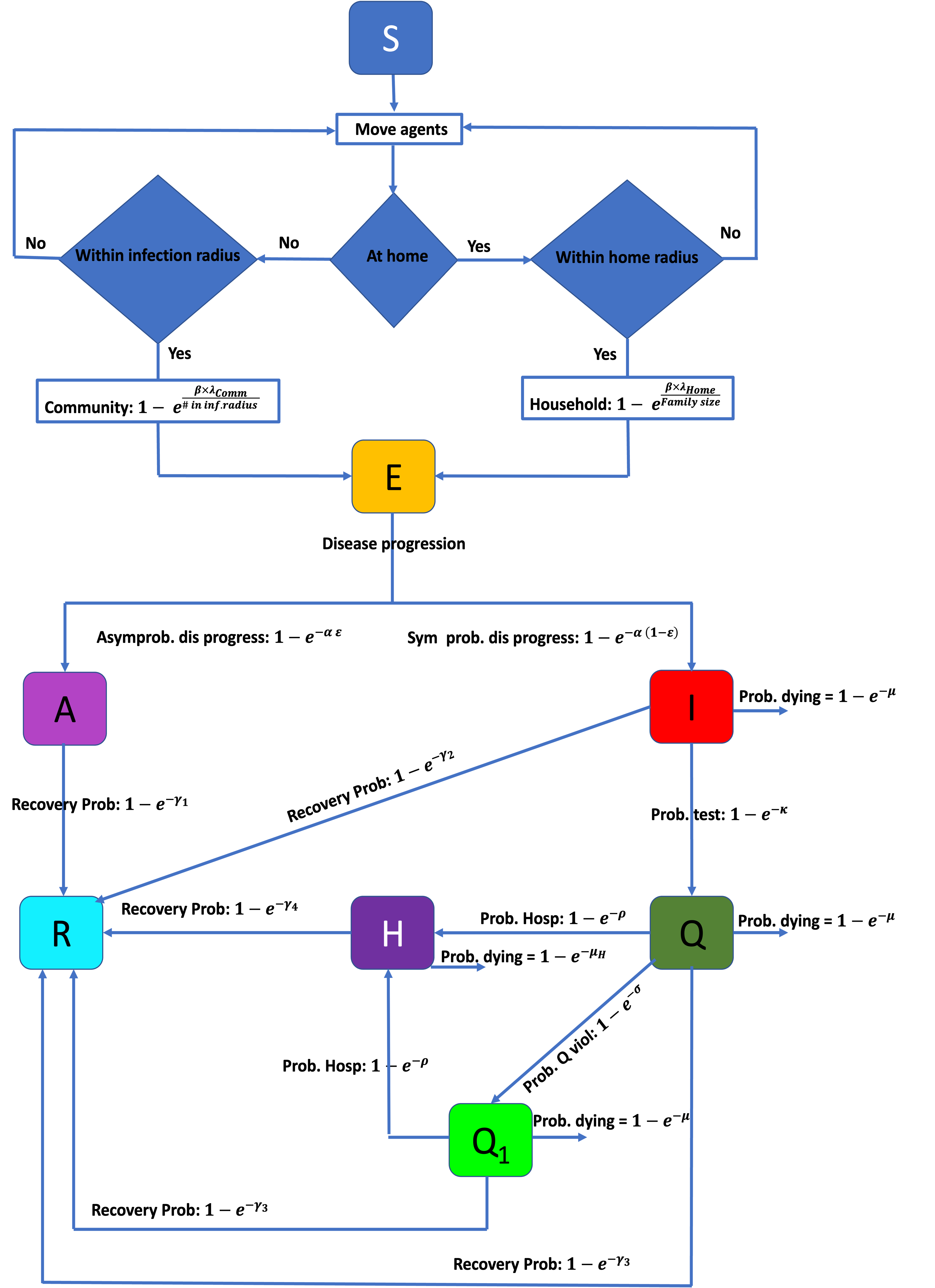

Supplement: S1 Fig — With this diagram we display the specific probabilities used by the Python code for the ABM. (TIFF) [file pone.0325619.s002.tiff]
